# Governance reforms for Pakistan’s National Institute of Health: Addressing challenges in disease surveillance and emergency management: A qualitative study

**DOI:** 10.1371/journal.pgph.0006231

**Published:** 2026-04-01

**Authors:** Babar Tasneem Shaikh, Zurva Ashraf, Mariam Ashraf, Zeeshan Iqbal Baig, Mumtaz Ali Khan, Muhammad Salman

**Affiliations:** 1 School of Public Health, Health Services Academy, Islamabad, Pakistan; 2 National Institute of Health, Islamabad, Pakistan.; Aga Khan University, PAKISTAN

## Abstract

The Centers for Disease Control in National Institutes of Health (CDC-NIH) in Pakistan is mandated to implement the Integrated Disease Surveillance and Response System (IDSRS) as well as a Public Health Emergency Management System (PHEMS) across the country for global health security and IHR-2005 compliance. This study aims to identify challenges in implementing IDSRS and PHEMS, explore the role of CDC-NIH as a leading public health agency, and propose solutions to improve coordination and governance. A qualitative approach was employed and data was collected through interviews. A rapid thematic content analysis approach was used for data analysis. The findings reveal that Pakistan’s public health system faces substantial challenges, particularly in implementing the IDSRS and managing public health emergencies. These constraints are deeply rooted in structural factors including: decentralization, which disperses authority across various levels; fragmented governance, leading to coordination problems; resource constraints that limit effective response; and legislative gaps that hinder policy implementation. These governance and legislative factors significantly impede Pakistan’s ability to respond efficiently to crises in the health system. Addressing these issues requires formalizing coordination mechanisms to ensure seamless collaboration and communication among stakeholders, clarifying governance arrangements to delineate roles and responsibilities, and establishing strong legal frameworks that provide a secure and enforceable foundation for actions. The strategic evolution of the CDC-NIH into a central coordinating authority could lead to a fundamental shift in public health governance, positioning it as a vital investment in national security, human development, and Pakistan’s health system resilience.

## Introduction

National Public Health Agencies (NPHAs) play a vital role in ensuring both national and global health security by coordinating functions to prevent, detect, and respond to public health threats and emergencies. The COVID-19 pandemic exposed major gaps in the way NPHAs operated, emphasizing the need for their restructuring and reform. The governance and structural frameworks of NPHAs determine how effectively and efficiently these agencies prepare for and respond to public health emergencies, thereby linking global health security to the strength of each NPHA’s governance model [1]. Understanding governance structures, encompassing both the formal regulations and informal norms that shape NPHA operations, as well as the agencies’ positions in relation to the Ministry of Health, the broader national public health system, and other key stakeholders and development partners is therefore essential for identifying the forces that influence their autonomy, accountability, and coordination capacity [2]. Such insights can, in turn, inform evidence-based restructuring and reform efforts aimed at enhancing NPHAs’ overall capacity.

The scope of NPHAs has expanded in recent years beyond communicable diseases to include non-communicable diseases, zoonoses, antimicrobial resistance, and climate-related threats [3,4]. To ensure that NPHAs are equipped to meet the demands of this broadened mandate, it is essential to examine lessons learned from recent public health emergencies to guide strengthening efforts for these agencies. For example, the COVID-19 pandemic highlighted the vital role of NPHAs while simultaneously exposing significant gaps in their legal frameworks and organizational structures, such as rigid procedures, limited autonomy, and unclear roles, which hampered dynamic and timely crisis response [2]. Drawing on the example of Pakistan, this study seeks to better understand the constraints faced by NPHAs and to outline strategies for strengthening governance and coordination mechanisms.

Pakistan’s complex federal structure, especially after the 18th constitutional amendment of 2010, has posed significant challenges for coordinating public health at the national level. The primary aim of this study is to examine the governance reforms needed at the CDC-NIH to enable effective national implementation of IDSRS and PHEMS. To examine and address these implementation challenges, in this paper we aim to answer three research questions: i) What are the challenges to implementing IDSRS and PHEMS in the federal context of Pakistan? ii) How can the coordination, governance, and legislative issues surrounding disease surveillance and health emergency management be addressed at the national and provincial level to enhance Pakistan’s public health emergency response capabilities?, and iii) How can the CDC-NIH’s role as the lead public health agency in Pakistan be further evolved to strengthen coordination, governance, and response to public health threats at national and provincial levels?

## Background: Pakistan’s Public Health Landscape

Pakistan is divided into four provinces and two federating areas, each with administrative and financial autonomy. The 18^th^ constitutional amendment, passed in 2010, devolved the administrative and financial powers, from the federal government to the provinces. The motivation for this reform was to dismantle the ‘presidential system’ that had been introduced by previous dictatorships, restore the parliamentary form of government, and implement greater decentralization by transferring more powers from the federal government to the provinces. All the vertical programs – mostly donor funded and managed by the federal tier of the government, were also devolved to the provincial health authorities [5]. Henceforth, data sharing from these programs, which are now managed by the provinces, has become a challenge. Further, the national surveillance system is ineffective due to unclear roles and difficulties in cross-provincial data sharing [6]. This decentralization creates tension between provincial autonomy and the need for national coherence in public health security, especially for trans-provincial threats. Further challenges emerged after the re-establishment of the Federal Ministry of Health in 2012 and ambiguities related to the mandate and role of the National Institute of Health (NIH). A subsequent National Institute of Health (Re-organizing) Act in 2021 restructured the entity into seven independent divisions, including the Center for Disease Control (CDC), mandating functions like disease surveillance, workforce development, and public health emergency management [7]. The country’s Joint External Evaluations in 2016 & 2023 highlighted gaps in demonstrating the sustainability of comprehensive surveillance systems and health emergency management capabilities [8,9]. The constitutional reform, while strengthening provincial autonomy in health governance, also created new tensions between decentralized administration and the need for nationally coherent public health security, particularly for trans-provincial threats.

The CDC in NIH plays significant role in implementing and operationalizing the Integrated Disease Surveillance and Response system (IDSRS) in Pakistan. The CDC sets the strategic agenda and mandates for infectious disease surveillance, ensuring that systems like IDSRS are effective in monitoring and responding to outbreaks. Meanwhile, the NIH serves as the custodian of disease surveillance & response, acting as the focal point for International Health Regulations (IHR) and overseeing the implementation of strategies to improve disease surveillance and data collection within Pakistan [10]. Diagnostic capacity is often limited, with shortages of laboratory equipment and trained personnel, leading to reliance on less specific syndromic surveillance. Overall laboratory infrastructure is inadequate, causing delayed case and outbreak confirmation. IDSRS provides a framework for strengthening the surveillance, response, and laboratory core capacities required by the International Health Regulations (IHR) 2005, a legally binding agreement providing a framework to coordinate and manage public health threats [11]. A strong national surveillance system is the foundation for global public health security and critical for response to epidemics, pandemics, and other emergencies [12].

Initially, IDSRS was piloted in eight selected districts of Pakistan for eight priority diseases. After completion of the pilot phase, a phase-wise approach for the implementation was adopted. During first phase, IDSRS has been implemented in more than 6000 primary and secondary healthcare facilities in all districts across the country, and more than 15000 healthcare personnel have been trained on disease surveillance. The DHIS-2 software/platform has been used to capture, analyze and store surveillance data. During future phases, the program will be expanded to the tertiary care level of public sector hospitals and subsequently to private health facilities. Moreover, public and private sector laboratories will also be integrated. The integration of vertical programs and other sectors (under One Health) will also be undertaken to establish a comprehensive and sustainable system for the country [13]. However, establishing effective coordination mechanisms for information sharing, outbreak response, and standardized protocols among provinces and with NIH, has proven challenging.

In addition to the implementation of IDSRS, CDC-NIH has initiated the process of developing a Public Health Emergency Management System (PHEMS) across the country in collaboration with the provincial health department and other relevant stakeholders [14]. The effectiveness and sustainability of both these initiatives depend upon coordination, governance, political commitment and ownership at all levels. There are also data-related challenges that limit effectiveness. Technological limitations, including inadequate Information and Communication Technology (ICT) infrastructure, hinder real-time data collection and sharing. There is minimal integration of advanced technologies, such as big data analytics and AI, for early detection. Community-Based Surveillance (CBS) programs are limited and not fully integrated into national systems. These deficiencies are interconnected, forming a cycle that prevents robust surveillance and emergency response [15]. Addressing Pakistan’s complex public health challenges requires a comprehensive, integrated approach that combines structural reforms, technological advancements, enhanced governance, and sustained investment [16].

## Materials and methods

### Ethics statement

This study obtained ethics review and approval from the Health Services Academy Ethical Review Board [No.7-82/IERC-HSA/2022–115]. Permission to conduct the research was also obtained from the National Institute of Health. All participants provided written informed consent to participate in the study. Interviews were recorded, ensuring anonymity and confidentiality of the participants.

This study was conducted in four provinces, Punjab, Sindh, Khyber Pakhtunkhwa, Balochistan, as well as Islamabad, the Capital Territory.

### Study setting

This was a descriptive qualitative study comprising in-depth key informant interviews (KIIs) with 37 key stakeholders across the provinces and at the national level. These interviews were strategically conducted across all administrative tiers to ensure comprehensive and representative data acquisition. A purposive sampling technique was meticulously implemented to identify and select key informants who possess specialized knowledge and insights pertinent to the research objectives. The sampling frame was developed through a structured stakeholder mapping exercise involving: (i) a desk review of institutional mandates and organizational charts of federal and provincial health bodies; and (ii) expert nomination by the research team and an advisory group. Stakeholders were categorized into five groups: (1) federal ministries and agencies (Ministry of National Health Services, NIH, CDC); (2) provincial health departments; (3) development partners (WHO, UNICEF, UKHSA); (4) non-governmental organizations; and (5) academic and research institutions. Across the four provinces and Islamabad Capital Territory, a total of 58 potential key informants were initially identified, of whom 37 consented and participated. The distribution by province/tier is as follows: Federal level (n = 9), Punjab (n = 8), Sindh (n = 7), Khyber Pakhtunkhwa (n = 7), Balochistan (n = 6). A detailed summary of participant characteristics, including institutional affiliation, designation, and administrative level, is provided in S1 Text.

### Study design

Data collection, management and analysis

The data collection, management, and analysis process in this study was designed to ensure comprehensive, accurate, and reliable results. The approach was structured to maximize the richness and validity of the data, and to ensure data triangulation by interviewing stakeholders at different hierarchies. The data collection took place from May to July 2025. Respondents included the federal health ministry, provincial health and livestock departments, NGOs, and development partners. The data was stored securely, with access limited to authorized personnel only. A systematic deductive coding process was employed to organize the data into themes and sub-themes, facilitating efficient retrieval and analysis. Data analysis was conducted using a rapid thematic approach, which involved identifying key themes and patterns within the data using the Policy Triangle as a guiding framework. This approach allowed for a comprehensive understanding of the challenges and opportunities in enhancing Pakistan’s public health emergency response capabilities.

### Inclusivity in global research

Additional information regarding the ethical, cultural, and scientific considerations specific to inclusivity in global research is included in the S1 File.

### Reflexivity

Several members of the research team are affiliated with the Health Services Academy and the National Institute of Health, institutions directly involved in the subject matter of this study. This insider positioning provided deep contextual knowledge of the policy environment, but also carried the potential for positional bias. To manage this, the following measures were employed: (i) structured interview guides were developed to minimize leading questions; (ii) all participant data were anonymized during analysis; (iii) findings were interpreted through iterative collaborative discussion among co-authors from different institutional backgrounds; and (iv) peer consultation was sought during data analysis and manuscript preparation. The authors acknowledge that, despite these measures, their institutional proximity to the research subject may have influenced the framing of analysis and recommendations.

## Results

The findings are based on interviews with key stakeholders involved in Pakistan’s Public Health system, specifically focusing on IDSRS and PHEMS. The analysis is organized around predefined themes and subthemes, using anonymized quotes to illustrate roles, challenges, barriers, and suggestions for enhancing federal-provincial coordination and governance. The results explicitly address the three research questions, providing a detailed overview of challenges, potential solutions, and the critical role of CDC-NIH in shaping Pakistan’s public health future (S2 File).

**Q1. What are the challenges to implementing IDSRS and PHEMS in the Federal Context?**

The implementation of IDSRS and effective PHEMS in Pakistan encounters a complex range of challenges. These difficulties are deeply embedded in the country’s federal structure, resource constraints, and systemic inefficiencies, collectively hindering a strong and unified public health response.

i Decentralization and Ambiguity of Roles and Ownership

All study participants shared that the most common challenge around decentralization is confusion about ownership and responsibility across different levels of the government health system. Delegating health services to the provinces was intended to empower local governance, but instead, it has unintentionally created a lack of clarity in defining specific roles between federal and provincial bodies. This structural change hinders efficient data collection, analysis, and coordinated efforts across the country. The participants shared that such a situation highlights a fundamental structural challenge where the pursuit of provincial autonomy inadvertently fragments the nation’s public health response.

**The 18**^**th**^ **Amendment has decentralized health governance, but it has also created ambiguities due to unclear legal authorities.** *MoNHSRC*

Common challenges in multi-tier systems include coordination gaps and resource disparities. Although policies such as the National Action Plan display important strengths, deficiencies in legal frameworks and data integration continue to hinder system performance. Furthermore, data-sharing and leadership gaps contribute to coordination challenges, while the NIH reorganization act contributes to additional uncertainty and ambiguity in roles.

**The Reorganization Act has created some gaps in coordination and clarity of roles, particularly in integrating new system.** *DOH, Sindh*

Legal gaps include the lack of mandates for direct reporting and inadequate emergency funding policies. Suggestions for addressing these gaps include developing a national Electronic Logistics Management Information System (e-LMIS), establishing task forces, and defining clear roles for federal, provincial, district, and international actors.

ii Weak Federal Leadership, Fragmented Systems, and Perceptions of NIH Effectiveness

Participants indicated that the federal government’s role in disease surveillance and emergency response is regarded as crucial but underdeveloped, calling for centralized leadership to standardize systems and enable swift action. Participants proposed that the federal government should lead by creating a national surveillance framework, establishing a centralized data platform/MIS, and providing training to strengthen provincial capacities.

**The federal government should play a central role in standardizing surveillance systems and ensuring data sharing across provinces.** *DOH, Balochistan*

In emergency response, the federal government is seen as a coordinator, ensuring resource allocation and standardized protocols through a national task force.

**A stronger NIH-CDC role could enhance data accuracy and lead to more effective public health management.** *DOH, Punjab*

However, stakeholders warned against over-centralization, which could undermine provincial autonomy.

**We must be wary of over-centralization, which could limit provincial autonomy.** *DOH, Punjab*

Participants suggested that the ideal federal-provincial relationship for disease surveillance and response involves a collaborative model where provinces maintain operational autonomy but follow a national framework, supported by digital platforms and regular task force meetings.

**We need to involve stakeholders at local, provincial, and federal levels to ensure comprehensive public health responses.** *MoNHSRC*

Based on participant responses, the federal level bodies, notably the NIH and the CDC, are not consistently perceived as strong nor reliable partners in disease surveillance and response. This perception often results in provinces operating independently, with little coordination and reliance on informal, ad-hoc channels.

**Coordination between federal and provincial levels relies on informal channels rather than a robust, formalized system, creating delays.** *DOH, Punjab*

The process of developing, implementing, and monitoring disease surveillance and emergency response involves multiple levels of governance. At the federal level, policy formulation and oversight are key, but implementation predominantly occurs at the provincial level, with districts executing on-the-ground activities. Federal roles are primarily supervisory, with NIH and CDC responsible for data analysis and international reporting; however, federal leadership in coordinating provincial efforts remains limited.

**There is a lack of effective unity between federal health agencies, which function separately despite being housed in the same premises** *. MoNHSRC*

**For effective collaboration, it’s crucial to establish clear roles, responsibilities, and mandates at both levels (Federal and Provincial).** *DOH, KP*

iii Resource Constraints (Financial, Human, Infrastructure)

Participants reported that both federal and provincial levels consistently face significant funding shortages, which severely impact their ability to manage emerging diseases, to sustain advanced testing facilities such as Biosafety Level-3 (BSL-3) laboratories, and to maintain the long-term viability of public health programs. The participants were of the view that the IDSRS, for example, often functions in ‘project mode,’ heavily dependent on donor support rather than being integrated into the regular health budget, which weakens its future stability.

**The IDSRS is still working on the project mode; however, no genuine efforts have been made to regularize it. There is a need for advocacy for the higher authorities.** *DOH, KP*

Participants shared that human resource instability, characterized by frequent transfers and periodic gaps in trained personnel, further disrupts continuity of care and increases reliance on external consultants and technical assistance from development partners. When donor funding ends or external consultants leave, these programs often weaken or collapse. This hinders the development of a self-reliant system as well as the retention of institutional memory, making long-term planning and resilience particularly challenging.

**The high burden of disease, such as Hepatitis C, TB, and polio, requires more resources. Resource constraints, both financial and human, are significant.** *Development Partner*

iv Budget Allocation

No dedicated, ring-fenced budget line exists for IDSRS or PHEMS within the national health budget. The programmes continue to operate largely in ‘project mode’ funded by development partners (WHO, USAID, UKHSA). Participants from development partner organizations estimated that external donor funding accounts for over 70% of operational costs for IDSRS. Without mainstreaming into the Public Sector Development Programme (PSDP), long-term sustainability is critically at risk.

v Technology, Data Sharing Gaps and Inefficiencies

Participants highlighted significant gaps in ICT infrastructure, particularly in rural and remote areas. DHIS-2, the platform used for IDSRS, functions reasonably well in urban settings but faces connectivity barriers at district level in Balochistan and southern KP.

At present, provinces share data with the NIH, either periodically or on an as-needed basis. Participants emphasized the absence of a centralized, standardized system for real-time data integration, resulting in significant delays and inefficiencies. In addition, provincial laboratories often report through complex hierarchical channels before data reaches federal entities, further slowing response times.

Participants reported that testing and reporting mechanisms for notifiable diseases, such as hepatitis and tuberculosis, are highly fragmented. Federal agencies are often unaware of the capacities within provincial laboratories, leading to a great deal of difficulty in data integration. They expressed the need for a national portal or standardized software, like a e-LMIS, for real-time data sharing.

**The absence of a unified national portal for IDSRS limits real-time surveillance.** *DOH, Sindh*

Results also suggest that information systems related to animal disease and surveillance are implemented through provincial systems such as Punjab’s Animal Disease Reporting System (ADRS), which uses mobile applications for real-time reporting across all districts.

**The ADRS system facilitates real-time reporting and response, with extension staff and mobile teams addressing field-level issues promptly.** *Livestock, Punjab*

In general, participants note that a one-way flow of information is keeping the system vulnerable to public health threats. It is challenging for provincial health departments to understand and address the gaps or improve their surveillance systems.

vi Underlying Public Health Determinants and Health Disparities

Participants highlighted poor hygiene, inadequate sanitation, and a lack of access to clean water as pervasive issues that contribute significantly to the disease burden in communities. These basic deficiencies exacerbate the challenges faced by disease surveillance, response, and emergency management efforts. Moreover, substantial health disparities exist between urban and rural areas, mainly because of limited access to healthcare services in rural regions. As a result, effective surveillance and response are particularly challenging in vulnerable populations, where outbreaks spread rapidly and disproportionately impact these communities with limited healthcare infrastructure.

**Q2. How can the coordination, governance, and legislative issues surrounding disease surveillance and public health emergency management be addressed at the national and provincial level to enhance Pakistan’s public health emergency response capabilities?**

Participants suggested that a multifaceted approach is needed that simultaneously concentrates on strengthening coordination mechanisms, clarifying governance structures, and reforming legislative frameworks. These interconnected areas are essential for enhancing Pakistan’s overall public health emergency response capabilities.

i Strengthening Coordination Mechanisms

As shared by all the participants, a fundamental shift is required from the current ad-hoc, relationship-based coordination towards formal, sustained mechanisms. This involves establishing clear communication channels, facilitating regular meetings between federal and provincial representatives, and implementing joint training programs to foster a culture of collaboration and mutual support. Continued reliance on case-by-case coordination without formal sustained efforts following major events like COVID-19 underscores the urgency of this transition.

**The federal government should lead in setting national policies and providing resources, while provinces implement localized responses.’** *Development Partner*

The successful model of the National Command & Operations Center (NCOC) during the COVID-19 pandemic is frequently cited as a prime example of effective coordination that needs to be institutionalized and sustained for all public health emergencies. The discontinuation of this mechanism after the immediate crisis highlights a lack of proactive strategic planning for long-term public health infrastructure. Participants suggested that establishing a formal forum for coordination at the policy level, potentially involving the Secretaries and Director Generals from both federal and provincial entities, is essential to maintain alignment and address emerging issues.

ii Clarifying Governance Structures and Defining Roles/Responsibilities

Participants shared that a foundational step to improving governance mechanisms is to clearly define roles, responsibilities, and mandates across federal, provincial, and district levels, to ensure effective collaboration, to prevent overlaps in service delivery, and to close existing gaps. This includes establishing dedicated focal points within provinces to improve coordination and response with federal entities.

**The response by the NIH is delayed, and we receive it when we have already acted in response to any emergency. For instance, Crimean-Congo Hemorrhagic Fever cases are more prevalent in Balochistan, necessitating financial and technical guidance on this issue; however, the response is consistently delayed.** *Livestock, Balochistan*

Participants emphasized that addressing the ‘verticality’ of different disease programs and surveillance streams is also critical. Many programs operate in silos, leading to significant duplication in disease reporting, such as malaria being reported on multiple platforms. A common platform is crucial for system integration, efficient use of human resource efforts, and avoidance of redundant activities.

iii Reforming and Harmonizing Legislative Frameworks

Participants widely agreed on the need for clearer legal authorities to define roles and responsibilities. A key recommendation is to develop a national Public Health Act that is applicable across all provinces and agreed upon collectively. Provinces would then update their respective provincial Acts to align with this national framework, ensuring the legal support necessary for a unified national health response. Participants suggested this legislative base as crucial for developing coherent policies similar to the ‘One Health’ approach.

Existing provincial Acts, such as in KP, show a provincial willingness to legislate on public health, participants highlighted that these examples also underscore the urgent need for harmonization, as some parts of provincial Acts may conflict with each other or lack clear provisions for federal coordination. For example, KP Public Health Act specifies coordination at the provincial level but lacks provisions for coordination with the federal government. Formal legal agreements, supported by political and legal mechanisms, are essential to ensure meaningful cooperation between federal and provincial health authorities.

**The IDSR governance system faces significant challenges as the chain of command is not clearly defined. While IDSR has been implemented, the governance framework and law enforcement situation remain weak.** *DOH, Balochistan*

**Q3. How can CDC-NIH’s role as Pakistan’s primary public health agency be further evolved to strengthen coordination, governance, and response at national and provincial levels?**

The interviews reveal a strong consensus on the crucial, yet currently underutilized, role of the CDC-NIH in leading Pakistan’s public health sector. Its development into a more resilient and central agency is seen as essential for enhancing national and provincial capacities in public health emergency response.

i Current Perceptions and Demonstrated Capacity

Participants noted that historically NIH has played a vital role, by providing essential services such as disease diagnosis through its reference laboratories, issuing timely advisories, and developing important guidelines. Its response during the COVID-19 pandemic, particularly through the NCOC, received international praise for effective coordination and feedback systems. Additionally, the NIH’s Field Epidemiology Training Program (FETP) is acknowledged as a major contribution, producing trained epidemiologists who are crucial in managing disease outbreaks across the country. Although these strengths were demonstrated, participants indicated the effective coordination mechanisms set up during COVID-19 were not maintained, leading to notable delays in tackling later outbreaks. They described the federal role as mainly focused on issuing guidelines and advisories, with little direct operational coordination with provinces.

**One major problem is the poor coordination between federal and provincial health authorities which has caused fragmented health governance.** *DOH, Punjab*

ii Ideal Role: Leading, Coordinating, and Capacity Building

Participants recommended that ideally, the federal government, through NIH and CDC, should take a leading role in creating a unified, integrated surveillance system linking all provinces. This would include establishing a national portal for real-time data sharing and adopting standardized reporting protocols across the country. Participants indicated that NIH-CDC should also serve as the main coordinator for rapid responses to public health emergencies, ensuring provinces have prompt access to essential resources, personal protective equipment and vaccines.

**The CDC-NIH proposed reorganization includes a separate institute for emergency management. The CDC has a strong role in coordination. At the federal level, NIH must boost its capacity, develop HR, and strengthen its systems.** *NIH*

**Public Health Emergency Response requires collaborative planning and resource sharing to ensure timely responses.** *Planning Cell, Balochistan.*

A stronger CDC-NIH role would involve defining its expanded mandate, actively supporting provinces with a comprehensive training program, building their capacity in key areas, and reinforcing existing systems. This would also require establishing a robust two-way communication and support system between NIH and provincial health departments.

iii Strategies for Enhanced Engagement and Sustainability

For the CDC-NIH to effectively develop into a leading public health agency, it should initially concentrate on enhancing its internal capacity, including human resources and systems. A model similar to the United States CDC, which shares expertise with all state health systems, was proposed by the participants as a feasible approach for Pakistan.

Participants emphasized that the provinces must be actively involved in decision-making processes and in co-developing the workplans to ensure that the federal strategies align with provincial needs and priorities, fostering a sense of ownership and commitment at the local level. They suggested establishing dedicated technical working groups at the national level, committed to consistent and active engagement rather than occasional consultations or meetings.

**The Federal role in Pakistan faces challenges such as limited resources, a lack of clear communication channels, and coordination issues between the federal and provincial levels.** *Planning Cell, Balochistan.*

### Key learnings and cross-cutting themes

Analysis of Pakistan’s public health system reveals several overarching themes that cut across the specific challenges and proposed solutions.

While decentralization successfully promoted provincial autonomy in managing their own health systems, participants noted that it inadvertently created a governance vacuum and significant coordination challenges at the federal-provincial interface.

A recurring observation is the ‘momentum loss’ phenomenon where successful ad-hoc coordination mechanisms, such as the NCOC during COVID-19, are not institutionalized as a sustained mechanism for public health emergencies management. This indicates a systemic tendency towards reactive, crisis-driven leadership rather than proactive investment in sustainable public health institutions.

The widespread ‘silo effect’ within the system, characterized by the vertical disease programs and fragmented data systems, was seen by the participants as an obstacle impeding holistic public health intelligence. This fragmentation not only leads to inefficiencies and duplication of effort but also prevents a comprehensive understanding of the overall health landscape, limiting the ability to identify wider epidemiological phenomena and to implement integrated interventions.

Participants also highlighted the ‘legislative lacuna’ (i.e., the absence of a comprehensive national Public Health Act and the lack of harmonization among the provincial Acts) as a critical barrier to unified action. Without a foundational legal framework that clearly defines roles, responsibilities, and coordination mandates, any progress in governance and coordination remains fragile and unsustainable.

Lastly, participants stressed the importance of a ‘partnership imperative’ for CDC-NIH’s evolution, suggesting that to truly become the leading public health agency, its success will depend on its ability to foster genuine partnerships with provinces through transparent processes, shared decision-making, co-creation of protocols, and mutual capacity building.

## Discussion

This study examined governance and coordination challenges facing the CDC-NIH in implementing IDSRS and PHEMS across Pakistan’s federal structure. Three overarching findings emerge. First, decentralization through the 18th constitutional amendment fragmented public health governance without establishing clear federal oversight mechanisms, creating a coordination vacuum between national and subnational health authorities. Second, the CDC-NIH lacks the institutional mandate, financial resources, workforce capacity, and digital infrastructure to serve effectively as the lead national public health agency. Third, the legal architecture, at both federal and provincial levels, is fragmented, with the absence of a comprehensive national Public Health Act representing a critical barrier to unified action. These structural deficits are compounded by a systemic tendency toward reactive, crisis-driven coordination (as seen with the NCOC model) rather than proactive, institutionalized public health governance.

The constitutional reform of 2010, while democratically justified in restoring parliamentary governance and devolving power from a historically over-centralized federal system, inadvertently fragmented Pakistan’s public health landscape. The study findings provide strong empirical support for the conclusion that re-centralization of coordination, not of service delivery, is now required to ensure national health security.

The governance challenges identified in Pakistan’s CDC-NIH are well documented across National Public Health Agencies (NPHAs) in the Global South and are supported by a robust evidence base for reform. Decentralization without legally defined intergovernmental roles consistently produces coordination vacuums and governance fragmentation. A realist synthesis across 25 countries, demonstrated that devolution frequently generates intra-jurisdictional inequities and coordination failures when constitutional mandates remain ambiguous, precisely the pattern observed in Pakistan following the 18th Amendment, where provinces operate independently and federal oversight remains largely advisory [16].

Nigeria’s experience in building the Nigeria Centre for Disease Control (NCDC) offers the most directly instructive parallel. The NCDC’s effectiveness required a clear legal mandate, investment in domestic workforce capacity through the Field Epidemiology Training Programme, and formalized sub-national coordination structures, none of which emerged through ad-hoc arrangements [17]. Pakistan’s CDC-NIH demonstrated strong capacity during COVID-19 through the NCOC, yet its post-crisis dismantlement reflects the same ‘momentum loss’ phenomenon: effective emergency mechanisms that were never institutionalized as sustained infrastructure. This reactive pattern, rather than proactive investment in permanent coordination structures, is a defining weakness of public health governance in decentralized systems across the Global South.

Resource dependency on donor financing further compounds institutional fragility. For instance, African public health systems’ reliance on international donors created critical vulnerabilities exposed during COVID-19, when shifting donor priorities weakened routine surveillance functions [18]. Pakistan’s IDSRS faces an identical risk by operating outside the regular health budget. A durable national public health response requires domestic financing and institutionalized workforce pipelines rather than perpetual dependence on external technical assistance [19]. Pakistan’s FETP represents a proven domestic asset that, if protected and expanded, can serve as a foundation for self-sustaining epidemiological capacity.

Taken together, the comparative evidence consistently demonstrates that governance reform, clarifying legal roles, institutionalizing coordination mechanisms, and securing domestic financing could yield disproportionately higher returns than isolated technical interventions. Strengthening CDC-NIH into a permanent central coordinating authority, underpinned by a National Public Health Act and standing operational structures, is the reform most strongly supported by global evidence.

One major limitation was the security situation in Balochistan, which required conducting interviews online instead of face-to-face. This may have affected the level of engagement and the depth of data collected from stakeholders in that region. Additionally, the research relies on qualitative interviews and may exhibit bias due to participants’ current roles and experiences.

### Limitations

Secondly, the study does not include healthcare workers at facility or community level, whose perspectives on surveillance operations would complement the policy and management views captured in the KIIs. This limits the ability to draw conclusions about implementation challenges at the frontline level.

While the findings of this qualitative study are deeply contextualized within Pakistan’s unique political and health system landscape, the challenges described are common to many countries in which major responsibilities related to global health security are devolved to subnational levels. The impact of such challenges has been noted in countries of all economic levels, for example, Nigeria and the United States [20]. An article in this supplement from the German Robert Koch Institute alludes to similar challenges. A striking finding from this study is the support of subnational interviewees for stronger, institutionalized national-subnational coordination and a robust NIH-CDC. Participants clearly saw a need to invest in governance reform (clarifying roles, processes, and legal frameworks), which may yield higher returns than isolated technical interventions. Thus, while the specific policy prescriptions are for Pakistan, the analytical framework and strategic approach can inform similar reform efforts globally.

### Considerations for Generalizability

This article is being published at a time when public health systems of many countries, especially those that are lower income, are changing rapidly due to cuts in donor aid. Examples of approaches countries are using to ensure critical surveillance and response functions are maintained include integrating vertical disease programs into routine health systems and increasing efficiency of surveillance and other core functions, including through the use of better information technology tools [21]. Addressing the kinds of challenges described in this paper will be critical for countries with decentralized systems to implement the kinds of changes that will ensure continuity and perhaps increase quality of surveillance and response.

Based on this research, several areas warrant further investigation. Firstly, a comprehensive financial analysis of the long-term costs associated with implementing and maintaining IDSRS and PHEMS at scale, including modelling of domestic financing scenarios. Secondly, a longitudinal assessment of governance reform implementation following any new national Public Health Act, to determine whether legislative changes lead to practical improvements.

### Policy implications for Pakistan’s Public Health System

The findings of this study carry several important policy implications:

1. Legal Reform is Foundational: Without a comprehensive national Public Health Act that provides clear mandates for federal-provincial coordination, any technical or governance intervention will remain fragile. The government should prioritize the passage of such an Act, working collaboratively with provinces to ensure alignment with provincial public health laws. Existing provincial acts (e.g., KP’s public health legislation) provide a useful starting point but require harmonization.
2. Institutionalize Coordination Beyond Crisis: The NCOC demonstrated that effective federal-provincial coordination is possible but requires deliberate institutionalization to survive beyond emergencies. A permanent, multi-sectoral Public Health Emergency Operations Centre (PHEOC) with a clearly defined mandate, permanent staffing, and a sustainable budget should be established.
3. Mainstream IDSRS and PHEMS into the National Budget: The current reliance on donor funding renders these programmes vulnerable to aid fluctuations. The government must develop a medium-term financing strategy that progressively shifts IDSRS and PHEMS operational costs to domestic budgetary allocations
4. Strengthen CDC-NIH’s Institutional Mandate: The NIH Reorganization Act 2021 created a structural basis for CDC-NIH’s expanded role. However, legislative mandate alone is insufficient without accompanying human resource development, budgetary allocation, and formal coordination protocols with provincial health departments.
5. Invest in Digital Public Health Infrastructure: Bridging the digital divide, particularly the connectivity gap in rural and remote areas, is a prerequisite for the national rollout of real-time surveillance. Universal access to DHIS-2 and, in the medium term, advanced predictive analytics tools should be treated as a public health investment rather than an IT cost.

## Conclusion

Pakistan’s CDC-NIH has the potential to become the country’s leading institution for coordinated public health initiatives, but this requires strategic transformation. Strengthening its mandate to create an integrated, nationwide surveillance and rapid response system is crucial. This must be accompanied by legal reforms, clear governance structures, and strong accountability frameworks that balance federal leadership with provincial autonomy. Sustained political commitment and investment are essential to implementing these reforms and building a resilient system that operates beyond emergencies. Enhanced coordination mechanisms, data integration, and capacity building, especially at provincial levels, will close existing gaps and foster shared ownership across all tiers. Ultimately, developing the CDC-NIH into a collaborative and empowered central authority can unify Pakistan’s fragmented health landscape, institutionalize preparedness, and establish a responsive and efficient public health system capable of protecting the nation against future health threats.

## Supporting information

S1 File Checklist: Inclusivity in global research. (PDF)

S2 File Data: Data Collection tool. (PDF)

S1 Text Province wise list of stakeholders interviewed. (DOCX)

## References

[1] Guerrero-Torres L, Shroff ZC, Hanefeld J, Lee VJ, Lynes S, Muvunyi C, et al. Governance and structure of national public health agencies: a critical foundation for strengthening public health security. BMJ Glob Health. 2025;10(5):e017724. doi: 10.1136/bmjgh-2024-017724 40441742 PMC12121559

[2] Rasanathan K, Hersey S, Shroff ZC, Namara G, National Public Health Agencies Governance Learning network. Governance of national public health agencies: a crucial yet neglected aspect of health emergency preparedness and response. BMJ. 2025;388:q2823. 10.1136/bmj.q2823 39792441

[3] Myhre SL, French SD, Bergh A. National public health institutes: A scoping review. Glob Public Health. 2022;17(6):1055–72. doi: 10.1080/17441692.2021.1910966 33870871

[4] World Health Organization. Strengthening health emergency prevention, preparedness, response and resilience. Geneva: World Health Organization. 2023.

[5] Government of Pakistan. 18th Constitutional Amendment, Constitution of the Islamic Republic of Pakistan. http://www.scribd.com/doc/30269950/18th-Amendment-in-the-Constitution-of-Pakistan-Complete-Text. Accessed 2025 October 10.

[6] Abdullah MA, Shaikh BT, Sikander A, Sarwar B. Public health and health system’s responsiveness during the 2022 Floods in pakistan: what needs to be done?. Disaster Med Public Health Prep. 2024;17:e567. doi: 10.1017/dmp.2023.224 38163991

[7] Gazette of Pakistan. The National Institute of Health (Re-organization) Act, 2021. Gazette of Pakistan. Islamabad: Government of Pakistan. 2021.

[8] World Health Organization. Joint external evaluation of IHR core capacities of the Islamic Republic of Pakistan. Islamabad: World Health Organization. 2016.

[9] World Health Organization. Joint external evaluation of IHR core capacities of the Islamic Republic of Pakistan. Islamabad. 2023.

[10] National Institute of Health. Functions of Center of Disease Control. https://www.nih.org.pk/center-for-disease-control. Accessed 2025 October 10.

[11] World Health Organization. International health regulations (2005). Geneva: WHO. 2005.

[12] Dsouza SM, Katyal A, Kalaskar S, Kabeer M, Rewaria L, Satyanarayana S, et al. A scoping review of health systems resilience assessment frameworks. PLOS Glob Public Health. 2024;4(9):e0003658. doi: 10.1371/journal.pgph.0003658 39312539 PMC11419368

[13] National Institute of Health. Integrated disease surveillance and response (IDSR) IDSR roadmap for Pakistan 2024-2028: A ‘system strengthening’ and ‘collaborative surveillance’ approach. Islamabad: National Institute of Health. 2024.

[14] National Institute of Health. Functions of Center of Disease Control. https://www.nih.org.pk/center-for-disease-control. Accessed 2025 October 10.

[15] Wolfe CM, Hamblion EL, Dzotsi EK, Mboussou F, Eckerle I, Flahault A, et al. Systematic review of Integrated Disease Surveillance and Response (IDSR) implementation in the African region. PLoS One. 2021;16(2):e0245457. doi: 10.1371/journal.pone.0245457 33630890 PMC7906422

[16] Abimbola S, Baatiema L, Bigdeli M. The impacts of decentralization on health system equity, efficiency and resilience: a realist synthesis of the evidence. Health Policy Plan. 2019;34(8):605–17. doi: 10.1093/heapol/czz055 31378811 PMC6794566

[17] Njidda AM, Oyebanji O, Obasanya J, Ojo O, Adedeji A, Mba N, et al. The Nigeria Centre for Disease Control. BMJ Glob Health. 2018;3(2):e000712. doi: 10.1136/bmjgh-2018-000712 29707246 PMC5914765

[18] Nkengasong JN, Mankoula W. Looming threat of COVID-19 infection in Africa: act collectively, and fast. Lancet. 2020;395(10227):841–2. 32113508 10.1016/S0140-6736(20)30464-5 PMC7124371

[19] Ihekweazu C, Agogo E. Africa’s response to COVID-19. BMC Med. 2020;18(1):151. doi: 10.1186/s12916-020-01622-w 32438912 PMC7242094

[20] Abubakar I, Dalglish SL, Angell B, Sanuade O, Abimbola S, Adamu AL, et al. The Lancet Nigeria Commission: investing in health and the future of the nation. Lancet. 2022;399(10330):1155–200. doi: 10.1016/S0140-6736(21)02488-0 35303470 PMC8943278

[21] World Health Organization. WHO issues guidance to address drastic global health financing cuts. https://www.who.int/news/item/03-11-2025-who-issues-guidance-to-address-drastic-global-health-financing-cuts. Accessed 2025 December 16.

