# Supplementary material for: Governance reforms for Pakistan’s National Institute of Health: Addressing challenges in disease surveillance and emergency management: A qualitative study
Source: PLOS Glob Public Health. 2026 Apr 1;6(4):e0006231. doi: 10.1371/journal.pgph.0006231 (PMC13042739; doi:10.1371/journal.pgph.0006231)
Supplement: S2 File — (PDF) [file pgph.0006231.s002.pdf]

Improving the Coordination and Governance at the National Institute of Health (NIH) for Enhanced Disease Surveillance and Emergency Management Across Pakistan

Key Informant Interview (KII) guidelines

A. Government partners at national, provincial and district-level stakeholders

General Interview Guidelines (for the interviewer)

The topics and questions below should be used to guide the interview but can be adapted as necessary for each interview. Keep in mind when interviewing to respond to the answers provided by the respondent by asking additional questions to those proposed below or adapting to more appropriate questions. Suggested probes are listed below each question, but add your probes as needed to ensure that the information provided is clear and to get more detail on topics mentioned that may be of relevance to the study.

Introduction

This study is being conducted to determine and document the challenges and issues with coordination, legislation and governance for disease surveillance of infectious diseases and outbreaks for health emergency management and to identify the possible solutions, including policy recommendations, to strengthen the coordination and governance mechanism. A qualitative study design is being employed covering the four provinces. Our focus is on the role of the federal level and federal-provincial relationships related to

These interviews will support in identifying the weaknesses, strengths, challenges and bottlenecks of the system and will guide the decision makers for possible solutions to improve and harmonize the governance mechanism at the country level for dealing with health emergencies of a larger scale. Subsequently, we envisage that this study will furnish the evidence necessary for a better governance model to be put in place for dealing with all sorts of health emergencies and to develop a resilient and responsive health system.

Introduction (guidelines for the Interviewer)

- Thank the participant for his/her time.
- Administer the informed consent procedure and proceed with the interview if the participant agrees to continue.
  - The participant should be provided with the opportunity to ask any questions.
- Before starting the interview, please explain to the participant:

Please note that we are interested in knowing your opinions. There are no right or wrong answers, but your opinions are important. Turn on the tape recorder, before starting the interview and after taking the consent

**Section I: General Information**	
Name of the participant/s	
Please specify the province or federal level	
Current Designation	
Highest Qualification	
Years of service in current role	
Organization	
Name of the Interviewer	
Date of Interview	
Duration of Interview	

**Section II**		
	**Question**	**Probe**
	**Context**	
**1.**	Please describe your current and past roles and experiences related to:<br>a. IDSRS?<br>b. Public health emergency response?	
**2.**	What are your observations about key issues or barriers to Pakistan's Related to the federal role?<br>- Related to federal-provincial relationships? Effectiveness in these areas?	
**3.**	4. What is your view of the role the federal government should play in:<br>a. IDSRS?<br>b. Public health emergency response?	

**5.**	What would a stronger NIH-CDC role look like?<br>a) What would be the potential benefits of a stronger NIH-CDC role?<br>b) What would be the potential negative impacts?	
**6.**	What is your view of how the federal-provincial relationship should work for:<br>a) IDSRS?<br>b) Public health emergency response?	
	**Content**	
**7.**	What are the factors that facilitate<br>o The federal level – NIH/CDC – efforts related to IDSRS? In emergency preparedness and response?<br>o Federal-provincial relationships related to IDSRS? Related to emergency preparedness and response?	o Probe for political, economic, cultural, and other factors
**8.**	What are the barriers that facilitate these?	
**9.**	What are the common challenges in multi-level policy systems in coordinating disease surveillance and public health emergency management both provincial and national?	
**10.**	What are the areas covered by the current policy and identify deficiencies, strengths, and gaps in coordination and decision-making specific to CDC-NIH	
	**Process**	
11.	What are the common coordination challenges at both the federal and provincial levels for:<br>a. ISDRS<br>b. Emergency response	
12.	To the extent it has been implemented, did the NIH re-organization create any new barriers or gaps in the ISDRS or managing public health emergency response?	
13.	Are there gaps in laws, regulations, policies, or other governance documents that would clarify or improve the federal role? Federal-provincial relationships?<br>o In IDSRS?<br>o In emergency response?	
	Process and Actors	
14.	Moving forward, what are your ideas about processes that should be used to address the gaps we have talked about related to IDSRS? To emergency response	
15.	Who would be the key actors in these processes?	
16.	Are the roles, responsibilities, and mandates for actors between federal, provincial, and district levels sufficiently clear?	
