# Supplementary material for: Governance reforms for Pakistan’s National Institute of Health: Addressing challenges in disease surveillance and emergency management: A qualitative study
Source: PLOS Glob Public Health. 2026 Apr 1;6(4):e0006231. doi: 10.1371/journal.pgph.0006231 (PMC13042739; doi:10.1371/journal.pgph.0006231)
Supplement: S1 Text — (DOCX) [file pgph.0006231.s003.docx]

## **Province wise list of stakeholders interviewed**

**Federal**

1. Director General, Ministry of National Health Services Regulations and Coordination
2. Country Lead UKHSA, Pakistan
3. Team Lead WHE, WHO Country Office Pakistan
4. Program Director, US-CDC country office, Pakistan
5. Chief, CDC NIH, Pakistan
6. Project Director, IDSR NIH
7. Team Lead, Public Health Emergency Operations Center, NIH
8. Director Programs, MoNHSRC, Islamabad

**Punjab**

1. Director General Health Services, Department of Health and Population, Punjab
2. Director, Communicable Disease and Epidemic Prevention & Control Program, Department of Health Population, Punjab
3. Integrated Disease Surveillance and Response Focal Person, Department of Health Population, Punjab
4. Incharge, Provincial Disease Surveillance and Response Unit, Department of Health Population, Punjab
5. Director MIS, Department of Health Population, Punjab
6. Laboratory Director, Provincial Public Health Laboratory, Punjab
7. Director General, Directorate General of Animal Health & Production Extension, Punjab
8. Chief Planning Officer, Planning Cell, Ministry of Planning, Development & Special Initiatives, Punjab

**Sindh**

1. Director General Health Services, Department of Health, Sindh
2. Director General, Sindh Institute of Animal Health, Karachi, Sindh
3. Additional Director, Provincial Disease Surveillance and Response Unit, Department of Health, Sindh
4. Deputy Director General, Communicable Disease, Department of Health, Sindh
5. Focal Person, Integrated Disease Surveillance and Response, Department of Health, Sindh
6. Additional Director, DHIS/ HMIS, DGHS office, Hyderabad, Sindh
7. Laboratory Director, Provincial Public Health Laboratory, Karachi, Sindh
8. Chief Planning Officer, Sindh Secretariat, Karachi

**Baluchistan**

1. Director General Health Services, Department of Health, Baluchistan
2. Director, Public Health, Department of Health, Baluchistan
3. Integrated Disease Surveillance and Response Focal Person, Department of Health, Baluchistan
4. Incharge, Provincial Disease Surveillance and Response Unit, Department of Health, Baluchistan
5. Laboratory Director, Provincial Public Health Laboratory, Fatima Jinnah General and Chest Hospital, Baluchistan
6. Director General, Directorate General of Animal Health & Production Extension, Baluchistan
7. Chief Planning Officer, Planning Cell, Ministry of Planning, Development & Special Initiatives, Baluchistan

**Khyber Pakhtunkhwa**

1. Director General Health Services, Department of Health, Baluchistan
2. Director, Public Health, Department of Health, Baluchistan
3. Integrated Disease Surveillance and Response Focal Person, Department of Health, Baluchistan
4. Incharge, Provincial Disease Surveillance and Response Unit, Department of Health, Baluchistan
5. Director General, Directorate General of Animal Health & Production Extension, Baluchistan
6. Chief Planning Officer, Planning Cell, Ministry of Planning, Development & Special Initiatives, Baluchistan
